# Determinants of Dietary Diversity Practice among Pregnant Women in the Gurage Zone, Southern Ethiopia, 2021: Community-Based Cross-Sectional Study

**DOI:** 10.1155/2022/8086793

**Published:** 2022-05-09

**Authors:** Tolesa Gemeda Gudeta, Ayana Benti Terefe, Girma Teferi Mengistu, Seboka Abebe Sori

**Affiliations:** ^1^Department of Nursing, College of Medicine and Health Sciences, Wolkite University, Wolkite, Ethiopia; ^2^Department of Midwifery, College of Medicine and Health Sciences, Wolkite University, Wolkite, Ethiopia

## Abstract

**Background:**

Dietary diversification is considered the proxy indicator of dietary quality and nutrient adequacy during pregnancy. Pregnant women have been considered susceptible to malnutrition because of their increased nutrient demands and thus consuming a variety of foods in their diet plays a lion's role in ensuring adequate nutrient intake. So understanding bottleneck factors associated with dietary diversity practice is very crucial to encouraging adequate dietary diversity practice. Therefore, this paper aimed to assess determinants of dietary diversity practice among pregnant women in the Gurage zone, Southwest Ethiopia.

**Methods:**

A community-based cross-sectional analytical study was conducted among 726 pregnant women, 13 key informants, and 27 focus group discussion *discussants* in the Gurage zone, southwest Ethiopia, from 1 September to 1 November 2021. A face-to-face interviewer-administered questionnaire was used to collect the data. According to the Minimum Dietary Diversity Score for Women (MDD-W) tool, women who consumed more than or equal to 5 of 10 food groups in the previous 24 hours had a diverse diet. Epi data version 3.1 was used for data entry, while SPSSversion 26 was used for analysis. To determine factors associated with dietary diversity, bivariate and multivariable logistic regression models were used to obtain crude odds ratio (COR), adjusted odds ratios (AOR), and 95 percent confidence intervals (CIs). Statistical significance was determined using adjusted odds ratios (AORs) with 95 percent confidence intervals (CIs) and *p* values less than 0.05. In narrative form, qualitative results were triangulated with quantitative data.

**Results:**

The overall prevalence of the adequate dietary diversity practice was found to be 42.1% with 95% CI (48.4–46.1%) and the mean dietary diversity score was 5.30 ± 1.49 standard deviation (SD). Multivariable analysis revealed that primary school level [AOR = 6.471 (2.905, 12.415)], secondary school level (9–12) [AOR = 7.169 (4.001, 12.846)], college and above level [AOR = 32.27 (15.044, 69.221)], women with higher empowerment [AOR = 3.497 (2.301, 5.315)], women with a favorable attitude toward dietary diversity [AOR = 1.665 (1.095, 2.529)], women from wealthier households [AOR = 2.025 (1.252, 3.278)], and having well-secured food status [AOR = 3.216 (1.003, 10.308)] were variables that influence dietary diversity practice. Three FGD and 13 key informant interviews were conducted, and the results of qualitative data generated three major themes.

**Conclusion:**

The overall prevalence of adequate dietary diversity practice was found to be low in this study when compared to studies conducted in Ethiopia. Maternal educations, mothers' attitudes toward dietary diversity, women empowerment, food security status, and wealth index level of the household were determinant factors that influence dietary diversity practice in this study. Therefore, programs aimed to improve pregnant women's dietary diversity practice should focus on improving the socioeconomic status and creating a congenial environment to promote women's empowerment.

## 1. Introduction

Pregnancy is a life stage during which tremendous anatomic, physiologic, biochemical, and metabolic changes have taken place to nurture the developing fetus and to prepare expecting women for labor and delivery [1]. These biochemical changes in turn change nutrition demand during pregnancy [2]. Nutrition during pregnancy is very important to meet the increased demands of energy, macronutrient, and micronutrient to support and maintain the health of the mother and growing fetus [3]. Thus, adequate diet during pregnancy supports adequate intrauterine growth of the fetus and normal birth weight, which can have a lifelong effect on development [4], and plays a vital role in the prevention of intergenerational effect of malnutrition (child born with low birth weight is more likely to have growth failure during childhood; in turn, girls born with a low birth weight are more likely to become small adult women) [5].

Multiple micronutrient deficiencies among pregnant women remain a major public health concern in low- and middle-income countries [3, 6, 7]. Malnutrition during pregnancy can permanently affect the physiological development of the fetus and increase the risk of intrauterine growth restriction, low birth weight, preterm delivery, and maternal morbidity and mortality [3]. Dietary diversification is the single most important strategy to address macro and micronutrient deficiencies during pregnancy [8]. Dietary diversity is described as the number of different foodstuffs or food groups consumed over a specific reference period [8–12], and it is a proxy indicator of dietary quality and nutrient adequacy during pregnancy and lactation [13, 14]. So pregnant mothers must eat different types of food groups which comprise sufficient quantity of minerals, protein, energy, vitamins, and water [8].

In low- and middle-income countries, pregnant women are predominantly at risk of micronutrient deficiencies. The majority of pregnant women in developing countries have inadequate nutrient intakes compared to the standard recommended by the World Health Organization (WHO) [3, 10] as their diets are monotonous, mainly cereal-based, and often include little, minimal consumption of nutrient-dense animal source food, fruits, and vegetables [10]. Two-thirds of pregnant women in developing countries are suffering from nutritional anemia [6]. South Asian countries accounted for 52.5% of anemia among reproductive women [15]. Also, sub-Saharan Africa and South Asian countries are suffering from the burden of micronutrient deficiencies such as zinc, iodine, and vitamin B12. It is noted that pregnant women are more liable for the risk of inadequate dietary intake than nonpregnant and nonlactating women [16].

Little studies conducted in Ethiopia have demonstrated that nutrient intakes of pregnant women are not adequate to meet their nutrient demand [17, 18]. According to the 2016 Ethiopian Demographic Health Survey (EDHS), 22% of women are thin, 24% are anemic, and 8% of women are obese due to inadequate dietary intake, limited diet diversity (vegetables and fruits), and changing lifestyles [19]. Inadequate dietary intakes are associated with intrauterine growth retardation, low birth weight, and premature delivery [20, 21]. These problems remained the main public health concern in Ethiopia and were associated with high neonatal and infant mortalities [19]. Conversely, excessive consumption of energy-dense foods is linked with excessive gestational weight gain [22, 23], which, in turn, increases the risk of adverse pregnancy outcomes [24].

Taking this into consideration, the Ethiopian government continuously showed its commitment to mitigating nutritional problems by developing food and nutrition policies, strategies, and programs. Furthermore, the government designed the “Seqota Declaration” to end stunting by 2030 [25]. Even though the effort of the government is in place to improve nutrition, few studies conducted on maternal nutrition in the country demonstrated suboptimal nutrient intakes of women during pregnancy compared with the standard recommended by the WHO [17, 18].

According to some studies conducted in the country, adequate dietary diversity among pregnant women varies within the country ranging from 12.8% in the Oromia region to 61.2% in the Tigray region [26–31]. Educational status of the mother, monthly household income, livestock ownership, women who received emotional support from their husbands, wealth index, women who engaged in shopping, and nutrition information are all factors that influence pregnant women's dietary variety practices [26–29]. However, research has scarcely investigated women's empowerment and women's employment in dietary diversity practice during pregnancy in Ethiopia. Although adequate dietary diversity practice is recommended and promoted, data on dietary diversity practice among pregnant women and its determinant factors in this study setting is limited. This calls for further investigations. Therefore, this study is aimed to assess determinants of dietary diversity practice among pregnant women in the Gurage zone, Southwest Ethiopia.

## 2. Methods and Materials

### 2.1. Study Area and Period

The Gurage zone is one of Ethiopia's administrative zones of the Southern Nation Nationalities and Peoples' Region (SNNPR). It is divided into 13 woredas and two administrative towns.

Wolkite town serves as the zone's capital. Wolkite is located 153 kilometers southwest of Ethiopia's capital, Addis Ababa. The Gurage zone now has a projected total population of 1,280,483, including 657,568 women, based on the 2007 census conducted by the Central Statistical Agency of Ethiopia (CSA). Since then, no further census has been done in Ethiopia [32]. There are 72 health centers and seven hospitals serving the zone's total population. Two of the hospitals in the zone are general zonal hospitals, while the other five are primary hospitals. The Gurage's main crop is Ensete, also known as Kocho (false banana plant). In addition to this, few cash crops (notably coffee, teff, and khat) and livestock (milk) are some sources of diet. In SNNPR, the percentage of women who received iron tablets for 90 days or more during recent pregnancy is lower (4.2%) which is lower than the national (5%) [33]. The study was conducted from 1 September to 1 November 2021.

### 2.2. Study Design and Population

The community-based mixed study design was employed from 1 September to 1 November 2021, among pregnant women residing in the Gurage zone. Quantitative data were complemented with qualitative data. The quantitative data collection method was followed by qualitative data collection to explore and explain the results of quantitative data results. All pregnant women in the Gurage zone were the source population, whereas the study populations were all the pregnant women in the selected kebeles (smallest administrative unit in Ethiopia). Since dietary diversity practice is affected by local culture, pregnant women who lived at least six months in the kebeles were included in the study. Pregnant women who had been diagnosed with and/or confirmed hypertension and/or diabetes mellitus were excluded from the study because these cases affect the dietary diversity practice of pregnant women.

### 2.3. Sample Size Determination and Sampling Procedure

The sample size for this study was calculated using Epi Info version 7 StatCalc function of sample size calculation for population survey at 95% confidence interval (CI), a margin of error of 5%, considering 31.4% of pregnant women had adequate dietary diversity practice from the related study conducted at Zille Tumuga district, Ethiopia [27], and adding 10% nonresponse rate gave us 363 study participants. However, due to the design effect, the final sample size for this study was 726. The design effect of 2 was used to calculate the sample size. For the qualitative part of the study, a minimum of 13 key informant interviews and three focus group discussions (FGDs) each group involving 6–12 participants were conducted based on the data saturation idea.

For quantitative data, a multistage sampling technique was employed. From 13 woredas and two town administrations in the zone, six woredas (Edegagn woredas (17 kebeles), Gomar woredas (18 kebeles), Enahor woredas (18 kebeles), Geta woredas (16 kebeles), East Meskan woredas (15 kebeles), South Sodo (17 kebeles)) and one town administration were selected by simple random sampling technique (SRST) using the lottery method. Then, four kebeles from each woreda and two kebeles from Wolkite town (6 kebeles) were selected by SRST again. House to the house (preliminary assessment) was conducted with the help of health extension workers (HEW) to identify eligible pregnant women in the selected kebeles (the total number of pregnant women identified was 1956). The sample size was proportionally distributed for each kebeles. Finally, a simple random sampling technique was used to select 726 pregnant women. The purposive sampling technique was employed for the qualitative part of the study.

### 2.4. Data Collection Tools and Procedure

A pretested and structured questionnaire was used to gather the data. An interviewer-administered questionnaire through the face-to-face interview was conducted at the participants' houses. The questionnaire consists of sociodemographic variables, obstetric and health-related factors, nutritional knowledge, dietary diversity practice, wealth index, food security status, and women empowerment status. The questionnaire was prepared in English and translated to the Amharic language and then retranslated to English by another person who is blind to the original questionnaire to check the consistency and flow of the questioners. Three Public Health Nutrition students and seven BSc nurses were employed as supervisors and data collectors, respectively.

The questionnaires for women empowerment were developed from Ethiopia's DHS [19] and other related studies [34]. The women empowerment index was assessed by asking the participants about women's involvement in household decision-making, membership in the community group, cash earning, ownership of household/land, and educational status. Finally, the score was summed and the women empowerment index was classified as low, moderate, and high. But during analysis, low and moderate groups were merged to create the dichotomous variable.

Dietary diversity measuring tools were adopted from minimum dietary diversity measuring 2016 FAO guidelines. The dietary diversity score of pregnant women was measured by asking the participants to list all food and drinks consumed in the past 24 hours. The documentation of each food was happen only when the consumed amount was greater than 15 g which was ensured using a food atlas [34]. Ten food groups were used, and food groups that consumed took “1” point, and those not consumed took “0”. The dietary diversity score was dichotomized into adequate dietary diversity (participants who consumed ≥5 food groups) and inadequate dietary diversity (participants who consumed <5 food groups) [35].

Tools measuring the wealth index of households were developed by Principal Component Analysis (PCA), and it was adopted from Ethiopian Demographic Health Survey (DHS) [36] and another related study [27]. More than twenty variables include household-related variables such as main fuel for cooking, availability of separate kitchen, possession of land, the main material of the wall, roof, and floor; certain household possessions were incorporated to measure the wealth index. Firstly, variables were coded between 0 and 1. After that, variables were entered and analyzed using PCA, and those variables having a communality value of greater than 0.5 were used to produce factor scores. After all, the factor scores were summed, and the wealth score was treated in tertiles for the analysis (poor, medium, and rich).

The food security status of the household was measured by Household Food Insecurity Access Scale (HFIAS) developed by Food and Nutrition Technical Assistance (FANTA) project [37]. It encompasses nine occurrence questions which represent a generally growing level of severity of food insecurity (access) and nine questions of “frequency-of-occurrence”. The frequency-of-occurrence question was jumped if the respondent reports that the condition labeled in the corresponding occurrence question was not experienced in the pasts four weeks (30 days). After all, the individual was considered as food secure if they respond “no” to all of the items or just experience worry but rarely if the household worries about not having enough food sometimes or often/or unable to eat preferred foods, mildly food insecure if household scarifies quality more frequently by eating a monotonous diet or undesirable foods sometimes or often, moderately food insecure, and severely food insecure if household experiences forced cutting back on meal size or the number of meals often and/or experiences any of the three most severe conditions.

The nutritional status of pregnant women was evaluated using nonstretchable measuring tape designed for adults' mid-upper arm circumference (MUAC). To ensure precision, the MUAC value of the left arm was measured to the nearest 0.1 cm with no clothes on the arm and in triplicate for each participant [38]. The participants were divided into two groups: undernourished (MUAC <23 cm) and normal (MUAC ≥23 cm) [39].

The Food and Agriculture Organization's (FAO) Knowledge, Attitude, and Practice Manual was used to develop nutritional knowledge assessment questions [40] and a cross-sectional investigation of pregnancy-related knowledge [41]. It has nine items, with a score ranging from 0 to 9. If a pregnant woman's knowledge score was 6 or above, she was categorized as having good knowledge, and if her score was lower than 6, she was labeled as having poor knowledge. Question on attitude toward nutrition during pregnancy is three points Likert scale with points ranging from “I don't think” to “yes I think”. Latifa et al. (2012) reported Cronbach's alpha of 0.82 [42]. The scale consists of 8 items measured with responses ranging from “I don't think” to “yes I think”.

### 2.5. Data Quality Management

To maintain the quality of the research, the questionnaire was prepared in English and translated to the Amharic language and then retranslated to English by another person who was blind to the original questionnaire to check its consistency. The questionnaire was adapted from a standard data collection instrument, and it was pretested. After the pretest, some modifications such as logical order and rewriting items that were difficult to understand were made and data collection time was estimated. The two-day (one-day theoretical and one-day practical) training was given to both the data collectors and the supervisors regarding the objective of the study, data collection tools, and procedures, in what way to approach respondents, and in what way to preserve confidentiality. The questionnaire was checked for completeness and consistency by the principal investigator and supervisors at the end of each day of data collection.

Finally, each questionnaire was entered into the software for analysis after being checked for data completeness and properly coded with a unique identification number. EpiData was employed for data entry since it has a monitoring mechanism for error detection. To cross-check data input uniformity, two different data clerks completed double data entry. Data was kept in the form of a file in a secure place where no one can access it except the principal investigator. For missing values and outliers, simple frequencies and cross-tabulation were done. This was verified by reviewing hard copies of the collected data.

For the qualitative part of the study, credibility was assured through long-lasting engagement with key informants and FGD discussants by investing sufficient time to become familiar with the setting and context, for misinformation testing, trust-building, and recognizing the data to get rich. Key informants and participants who were genuinely willing to take part and prepared to offer data freely were involved in the study. Transferability of the study was secured by describing the behavior and experiences of the participants with their context; hence, the behavior and experiences become meaningful to an outsider. The dependability and conformability of the study were secured by clearly describing the research footsteps taken from the start of a research proposal to the development and reporting of the findings. Throughout the study, the records of the research path were kept.

## 3. Data Processing and Analysis

Data were first checked for completeness and consistency; the corresponding code number was written carefully at each margin, double entered into Epi data version 3.1, and then exported to Statistical Package for Social Science (SPSS) version 26 for analysis. In addition to descriptive statistics, both bivariate and multivariable logistic regression analyses were performed. Descriptive analyses were conducted by using frequency and percentages. In the bivariate logistic regression analyses, those variables with a *p* value <0.25 were entered into multivariable logistic regression models for controlling confounding factors. Multivariable logistic regression analysis that used a backward stepwise method of variable selection was applied to describe the relative effect of independent variables on dietary diversity practice among pregnant women. The adjusted odds ratio was used to determine the strength of association between many independent variables with outcome variables after controlling confounding factors. A multicollinearity test was carried out to see the correlation among the independent variables using the variance inflation factor. None of the variables had an inflation factor >10 (i.e., VIF <3.107 in this study). The goodness of fitness of the model was checked by Hosmer and Lemeshow assumption test (*p* value = 0.598 in this study) which indicates that the model was adequately fitted. Finally, statistical significance was declared at *p* value <0.05 at multivariable logistic regression.

### 3.1. Qualitative Data

Semistructured interviews and FGD guiding questions were used, and it was developed after reviewing different literature. The questions had two parts: sociodemographic characteristic-related questions and questions regarding dietary diversity practice during pregnancy. The semistructured interview and FGD guiding questions were checked by experts. The 13 key informants were health professionals, community leaders, pregnant women, and nutrition officers. Eleven FGD participants were pregnant women, nine were husbands, and seven were health professionals. During data collection in addition to the tape recorder, field notes were used. The in-depth interview (IDI) was conducted by the principal investigator in a silent and comfortable room after its time and place were decided by the interviewees. The interviews were carried out in Amharic (local language). The interviews were lasting 30 to 45 minutes. FGD was facilitated by the principal investigator also, and the FGD took from 45 min to 1 h. Notetakers who have experience in qualitative data collection were recruited and took notes during the In-depth interview and FGD of this study.

Interviews and FGD were transcribed verbatim into plain text, translated into English, then imported directly into ATLAS.ti.7.5.16 for coding, and analyzed thematically. So, each script was read carefully again and again, and then, codes were created by two data coders. Data relevant to each code were collected together and cross-checked between researchers. Then, through constant comparison of concepts, like with like, the codes were grouped into three major themes. It was checked whether or not the themes worked with the coded extracts. Lastly, the quantitative results and illustrations were triangulated and presented with qualitative results in narrative forms.

### 3.2. Ethical Consideration

Before data collection, the ethical clearance was obtained from the institutional review board (IRB) of the College of Health Science and Medicine, Wolkite University. An official letter was sent to the Gurage zone health office to get their permission. Data collection was begun after permission, and a cooperation letter was written to all districts in which the study was carried out. The purpose and significance of the study were explained to the study participants. Participants were asked for their voluntary participation, and they were informed to withdraw themselves at any time without giving any reason if they do not want to proceed. They were also informed that their information will be kept confidential. Interviews were recorded after permission was taken from participants. The privacy and identity of the participants were protected. Before agreeing to participate, the phone number and email address of the principal investigator were provided to help participants ask questions related to unclear aspects of the study or request for study results. Finally, written consent (fingerprints for women who could not read and write) and verbal informed consent were obtained.

## 4. Results

In this study, seven hundred twenty-two pregnant women were interviewed, making a response rate of 99.4%. The mean age of the study participants was 27.84 (SD ± 5.038). Out of the 722 respondents, 444 (61.5%) participants were within the age group of 25–35 years and 191 (26.5%) were within the age group of <25 years. Seven hundred four (97.5%) were married, and 313 (43.4%) were Islam in religion. Regarding education, the majority of the participants (64.0%) were illiterate. Approximately, half of the study participants were from poorer wealth status. More than half of the women were classified as low/moderately empowered and belonged to food-secure households (Table 1).

Three FGD and 13 key informant interviews were conducted, and the results of qualitative data generated three major themes. The three major themes that emerged from qualitative data were inadequate dietary diversity practice, facilitators and barriers of dietary diversity practice, and proposed solutions for dietary diversity practice (Table 2).

### 4.1. Reproductive History, Knowledge, and Attitude toward Nutrition-Related Characteristics of Study Participants

Approximately, one-fourth of the participants were multipara women, and half (47.1%) of the study participants were found in the second trimester. More than half (59.0%) of the participants had a greater than or equal to the two-year gap between pregnancies. Approximately, all (96.3%) of the participants had a history of ANC visits during the current pregnancy. More than half of the participants were counseled on dietary intake. Approximately, half (48.1%) of the study participants had a desirable attitude toward nutrition during pregnancy. The qualitative result complimented this in that a 29-year-old participant said, “*pregnant mothers are very hesitant in preparing and consuming diversified foodstuffs, they choose the consumption of monotonous food. They prepare certain food in huge and consume it for one to two days in parallel*” (Participant 15). More than half of the participants had adequate (good) knowledge about nutrition during pregnancy. This finding was corroborated by the qualitative finding as 13 key informants and 17 FGD discussants reported a lack of knowledge on diet during pregnancy as a bottleneck factor for adequate dietary diversity practice. A nurse key informant government employee reported, “I *have no adequate information on maternal nutrition during pregnancy, thus way I give less emphasis for nutrition counseling compared with other packages*…..*the discussant also added that in this area pregnant women are less knowledgeable on maternal nutrition during pregnancy thus the way they sell their beneficial foodstuffs and consume grains during their pregnancy*” (Participant 12) (Table 3).

### 4.2. Dietary Diversity Practice Status

**Figure 1:**
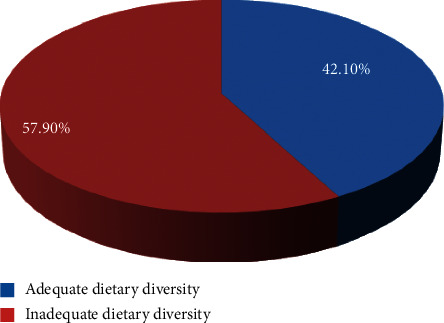
Dietary diversity practice among pregnant women over the last 24 hours in Gurage zone southern Ethiopia 2021 (*n* = 722).

The overall prevalence of the adequate dietary diversity practice among pregnant women in the Gurage zone was found to be 304 (42.1%) with 95% CI (48.4–46.1%) (Figure 1).

The mean dietary diversity score was found to be 5.30 with SD ± 1.49 in this study. This finding was corroborated by the qualitative finding. Even though all participants reported the importance of consuming a diverse diet during pregnancy, most of the respondents in key informant interviews and FGDs reported inadequate dietary diversity practices in pregnant women. A 26-year-old pregnant FGD discussant noted that “*I personally frequently eat Enset or Kocho (false banana plant) with cabbage, the discussant added that however, the frequency of the intake is less during pregnancy compared with intake before pregnancy by considering that consuming many times predispose to a big baby as the community suggests*” (Participant 25).

The majority of the participants (89.5%) consumed grains, white roots, and tubers, whereas 100% of the participants consumed pulses. From animal food sources, more than one-third of the study participants consumed milk and milk products (34.1%) and eggs (39.1%), and less than one-fourth of the participants consumed meat, poultry, and fish. This finding was corroborated by the qualitative finding. One main reason for poor dietary diversity practice is the habit of the practice of fasting exclusively among Orthodox religious followers. Ten key informants and almost all FGD discussants reported that “*Pregnant women in this area avoid consuming animal products and do not eat breakfast on fasting days. These prevent them from eating a balanced diet and make it very difficult for them to exercise nutritional diversity throughout pregnancy*”. Greater than one-fourth of the participants consumed dark green leafy vegetables, and only 23.7% consumed vitamin A rich fruit and vegetables. This finding was corroborated by the qualitative finding as one to five network leader key informant participants said that “*The Ensete, or false banana plant, plays a very important role in Gurage society's daily interactions and components of daily social and ritual life, and it is usually recommended diet for pregnant women*. *The Ensete's ritual values include covering the body with Ensete leaves after death, using leaves as a cord tie after birth, and covering food things with the corps, which is regarded blessed food in Gurage society*.”

*“Despite the fact that all foodstuffs are available in the home, pregnant women choose Kocho, a thick bread prepared from Ensete and cabbage that is regarded a traditional, blessed, and dignified cuisine in Gurage society*.” (Key informant participant 5). Consumption of other vegetables was reported by 92.2% of the participants whereas the consumption of other fruits was reported by less than one-fourth of the participants (Table 4).

### 4.3. Factors Associated with Dietary Diversity Practice During Pregnancy

The bivariate logistic regression model showed that the age of the participants, educational status, occupational status, household wealth index status, food security status, nutritional status, women empowerment status, getting health education on dietary diversity, knowledge, and attitude toward dietary diversity during pregnancy were found significantly associated with dietary diversity of pregnant women in the study area. In the adjusted model educational status, women empowerment, attitude toward dietary diversity, wealth index status, and food security status were significantly associated with the dietary diversity practice of pregnant women.

The odds of pregnant women who attended primary school [1–8] were 6.471 times [AOR = 6.471 (2.905, 12.415)], women who attended secondary school [9–12] were 7.169 times [AOR = 7.169 (4.001, 12.846)], and those who attended college and above level were 32.27 times [AOR = 32.27 (15.044, 69.221)] more likely consume diversified food compared with illiterate women. The likelihood of adequate dietary diversity practice during pregnancy was 3.497 times higher among women who reported experiencing higher levels of empowerment [AOR = 3.497 (2.301, 5.315)] than their counterparts. This finding was corroborated by the qualitative finding as a 30-year-old FGD discussant pregnant woman said that “*…I have my personal small business and regular income, making a successful living for my family by availing important food items*…..*the discussant also added her family's diets are better than her neighbors even though I don't know the reason*” (Participant 18). Pregnant women with a favorable attitude had 1.665 times higher odds of consuming diversified food than women with unfavorable attitudes [AOR = 1.665 (1.095, 2.529)]. Concerning wealth index, the odds of consuming adequate diversity dietary practice were 2.025 times [AOR = 2.025 (1.252, 3.278)] higher in pregnant women from rich households than the pregnant women from poor households. The qualitative finding supported this in that a 25-year-old female health care provider key informant interviews discussant participant said, “*People who have money can buy and eat various foodstuffs, but those with poor socioeconomic level cannot buy or afford them, even if food products are available in the market*. *This is especially true during the Covid-19 disease outbreak, when the price of food items continues to rise*.” (Key informant participant 2).

Similarly, this study also showed that women who had well-secured food were 3.216 times [AOR = 3.216 (1.5003, 10.308)], those with mildly food insecure were 2.431 times [AOR = 2.431 (.728, 8.118)], and those with moderate food insecure were 4.614 times [AOR = 4.614 (1.315, 16.190)] more likely consume diversified food than their counterparts. Most all qualitative study discussants reported that food insecurity is the main setback for adequate dietary diversity practice during pregnancy. A pregnant mother FGD discussant women reported, “*in this area, some pregnant women are living in poor living standards, some have large family size, for these pregnant women, food insecurity becomes an obstacle to have good dietary diversity practice*” (Participant 3) (Table 5).

## 5. Discussion

This study was carried out to assess the prevalence of adequate dietary diversity practice and its determinant factors among pregnant women in the Gurage zone. Understanding the determinants of dietary diversity practice of pregnant women is very important to tackle the intergenerational effect of malnutrition. This study indicates that the overall prevalence of adequate dietary diversity among pregnant women in the Gurage zone was found to be 42.1% (95% CI 38.4–46.1%). This is nearly similar to a study conducted in Ethiopia (45%) [43], Dire Dawa City, Eastern Ethiopia (43.0%) [44], East Gojjam zone, Northwest Ethiopia (44.3%) [43], Bale zone, Southeast Ethiopia (44.8%) [45], Northern Ghana (46.1%) [46], and Bangladesh (37%) [47] but lower than the study conducted in Tigray, northern Ethiopia (61.2%) [31], Kolfe Keranyo subcity health centers, Addis Ababa (60.9%) [28], Kenya [48], and Malawi [49]. The discrepancy in these findings might be attributed to the difference in study settings, sociodemographic characteristics of the study participants, and availability and accessibility of health service infrastructures. In this study, the majority of the participants were illiterate compared with the abovementioned pieces of evidence, which may affect the mother's knowledge and dietary diversity practice. However, the findings of this study are higher than some studies conducted in different corners of Ethiopia: 31.4% in the Amhara region [27], 25.4% in Oromia [50], and 20.1% in southern Ethiopia [51]. This difference might be due to the difference in the study setting and sociocultural characteristics.

The other main finding of this study was the identification of determinant factors associated with adequate dietary diversity practice. Accordingly, educational status, women empowerment status, household wealth index, food security status, and mother's attitude toward dietary diversity practice were significantly associated with the adequate dietary diversity practice.

The finding of this study indicated that the odds of having adequate diversified diets were higher among pregnant women who had attended primary, secondary, college, and above educational level than those who were illiterate. This finding was supported by the study finding from Northeast Ethiopia [27], Nigeria, Rural Bangladesh, Kenya, Nepal, and Ghana [34, 47, 48, 52–54]. One possible explanation is that educated women can get better employment opportunities and have a regular income which can further directly or indirectly improve the purchasing power of different foodstuffs and other agricultural inputs compared to illiterate pregnant women. On top of this, women with higher education might have learned crucial information on appropriate feeding practices. This highlighted that programs to improve women's nutrition should focus on empowering women through education.

Another factor that was found to be statistically significantly associated with adequate dietary diversity practice was women's empowerment status. The odds of adequate dietary diversity practice among pregnant women experiencing higher women empowerment were greater than three times higher compared to their counterparts. This finding is consistent with the study conducted in Kenya [48] and Nepal [34]. The possible explanation is the role of gender in determining dietary diversity practice. So women should overcome any gender-based obstacles to be a success in terms of diets and dietary diversity practices. Concerning the household wealth index, women from wealthier households had a greater chance of consuming diversified diets than women from poorer households. This finding is supported by studies done in Nepal [34], rural Bangladesh [55], and Kenya [48] as well as a study conducted in the Terai region of Nepal, which found that socioeconomic status was positively associated with more frequent consumption of most food groups including in-season fruits and vegetables [56]. One possible explanation is that higher/greater income is linked with improved purchasing power which in turn helps promote and maintain dietary diversity practice.

Another factor that was found to be statistically significantly associated with adequate dietary diversity practice was food security status. We found that pregnant women who had secured food have high dietary diversity than those who had unsecured food (severely food insecure). This is supported by the study conducted in the Tigray region, Ethiopia [31], and Malaysia [57] which showed pregnant mothers who had secured food status were more likely to have adequate dietary diversity practices than their counterparts. This study also revealed that attitude toward nutrition during pregnancy is significantly associated with dietary diversity practice. The odds of dietary diversity practice were 1.665 times higher among pregnant women with a favorable attitude than women with unfavorable attitudes. This finding is consistent with the study conducted in Tanta city [42].

This study used both qualitative and quantitative methods of data collection to maximize the reliability of the finding. However, the study has some limitations. First, the study relied on the memory of the participants about the food they consumed over the last 24 hours which may have introduced a recall bias. Second, because of the cross-sectional nature of the study, causality could not be explained. Finally, social desirability bias might be introduced during answering wealth index questions.

## 6. Conclusion

The study has demonstrated that about 42.1% of the study participants had adequate dietary diversity practice, whereas 57.9% had inadequate dietary diversity practice. The study also showed the critical role of maternal education, attitude toward nutrition, women empowerment, food security, and wealth index in attaining and maintaining adequate dietary diversity practice among pregnant women. The FGD and in-depth interview results also complemented this many of these findings. Therefore, interventions and public health policies targeted at promoting pregnant women's dietary diversity practice should focus on creating a congenial environment to promote women's empowerment and socioeconomic status, and health professionals trained in nutrition, and health extension workers should strengthen their effort in disseminating information regarding nutrition during pregnancy. A longitudinal study is advisable to establish cause-and-effect relationships.

## Figures and Tables

**Table 1:** Sociodemographic characteristics of the pregnant women, in the Gurage zone, southwest Ethiopia, September to November 2021 (*n* = 722).

Variable	Category	Frequency	Percent
Age in years	<25	191	26.5
	25–35	444	61.5
	>35	87	12.0

Marital status	Married	704	97.5
	Single	13	1.8
	Divorced/separated	5	.7

Religion	Orthodox	296	41.0
	Islam	313	43.4
	Protestant	81	11.2
	Catholic	32	4.4

Educational status	Illiterate	462	64.0
	Primary	42	5.8
	Secondary	90	12.5
	Collage and above	128	17.7

Occupational status	Government employed	122	16.9
	Merchant	230	31.9
	Housewife	289	40.0
	Students	81	11.2

Wealth status	Poor	354	49.1
	Medium	206	28.5
	Rich	162	22.4

Women empowerment	Low/moderate	456	63.2
	High	266	36.8

Food security status	Food secure	413	57.2
	Mildly food insecure	180	24.9
	Moderately food insecure	90	12.5
	Severely food insecure	39	5.4

Nutritional status	<23	259	35.9
	23 and above	463	64.1

**Table 2:** Sociodemographic characteristics of qualitative study participants in the Gurage zone, southern Ethiopia, September to November 2021.

Variable	Key informant interviews (*n* = 13)	Focus group discussion (*n* = 27)
Age in years		
≤25	4	7
26–35	6	14
≥36	3	6

Sex		
Female	8	15
Male	5	12

Educational status		
No formal education	2	14
Primary education	3	2
Secondary education	2	4
Diploma and above	6	7

Marital status		
Married	8	21
Unmarried	5	6

**Table 3:** Obstetric characteristics of pregnant women in the Gurage zone, southwest Ethiopia, September to November 2021 (*n* = 722).

Variables	Category	Frequency	Percent
Parity	Primipara	183	25.3
	<2	112	15.5
	2–5	401	55.5
	>5	26	3.6

Trimester	First trimester	37	5.1
	Second trimester	340	47.1
	Third trimester	345	47.8

Space between pregnancy (*n* = 539)	<2 years	221	41.0
	≥2 years	318	59.0

ANC visit	No	27	3.7
	Yes	695	96.3

Taking iron and folic acid	No	97	13.4
	Yes	625	86.6

Counseling of dietary intake during pregnancy	No	273	37.8
	Yes	449	62.2

Nutritional knowledge	Poor knowledge	242	33.5
	Good knowledge	480	66.5

Nutritional attitude	Undesirable attitude	375	51.9
	Desirable attitude	347	48.1

**Table 4:** Dietary diversity practice of study participants in the Gurage zone, southwest Ethiopia, September to November 2021 (*n* = 722).

Characteristics	Number	Percentage
Food groups		
Grains, white roots, and tubers	646	89.5
Pulses	722	100.0
Nuts and seeds	612	84.8
Milk and milk products	246	34.1
Eggs	282	39.1
Meat, poultry, and fish	126	17.5
Dark green leafy vegetables	212	29.4
Vitamin A rich fruit and vegetables	171	23.7
Other vegetables	666	92.2
Other fruits	170	23.5

Dietary diversity status		
Diverse % (95% CI)	304	42.1 (38.4–46.1)
Not diverse % (95% CI)	418	57.9 (53.9–61.6)
Mean ± SD	5.30 (±1.49)	

**Table 5:** Bivariate and multivariable logistic regression of factors associated with dietary diversity among pregnant women in the Gurage zone, southwest Ethiopia, 2021 (*n* = 722).

Characteristics	Dietary diversity		COR (95% CI)	AOR (95% CI)
	Inadequate (%)	Adequate (%)		
Educational status				
Illiterate	360 (77.9)	102 (22.1)	1	1
Primary (1–8)	15 (35.7)	27 (64.3)	6.353 (3.256, 12.395)^*∗∗*^	6.471 (2.905, 12.415)^*∗∗*^
Secondary (9–12)	33 (36.7)	57 (63.3)	6.096 (3.765, 9.870)^*∗∗*^	7.169 (4.001, 12.846)^*∗∗*^
Collage and above	10 (7.8)	118 (92.2)	41.647 (21.058, 82.365)^*∗∗*^	32.27 (15.044, 69.221)^*∗∗*^

Women empowerment				
Low/moderate	321 (70.4)	135 (29.6)	1	1
High	97 (36.5)	169 (63.5)	4.143 (3.007, 5.708)^*∗∗*^	3.497 (2.301, 5.315)^*∗∗*^

Attitude toward dietary diversity				
Unfavorable (score <75%)	233 (62.1)	142 (37.9)	1	1
Favorable (score ≥75%)	185 (53.3)	162 (46.7)	1.437 (1.068, 1.933)^*∗*^	1.665 (1.095, 2.529)^*∗*^

Wealth index				
Poor	255 (72.0%)	99 (28.0%)	1	1
Medium	107 (51.9%)	99 (48.1%)	2.383 (1.665, 3.411)^*∗∗*^	2.457 (1.570, 3.845)^*∗∗*^
Rich	56 (34.6%)	106 (65.4%)	4.876 (3.273, 7.262)^*∗∗*^	3.795 (2.299, 6.264)^*∗∗*^

Food security status				
Food secure	240 (58.1%)	173 (41.9%)	3.965 (1.626, 9.669)^*∗*^	3.216 (1.003, 10.308)^*∗*^
Mildly food insecure	104 (57.8%)	76 (42.2%)	4.019 (1.604, 10.073)^*∗*^	2.431 (.728, 8.118)
Moderately food insecure	41 (45.6%)	49 (54.4%)	6.573 (2.507, 17.231)^*∗*^	4.614 (1.315, 16.190)^*∗*^
Severely food insecure	33 (84.6%)	6 (15.4)	1	1

^*∗*^Statistically significant variables.

## Data Availability

All relevant data and supporting information files are within the manuscript. Additionally, the data sets used to support the findings of this study are available from the corresponding author upon request.
